# Supplementary material for: Enhanced Arctic sea ice melting controlled by larger heat discharge of mid-Holocene rivers
Source: Nat Commun. 2022 Sep 13;13:5368. doi: 10.1038/s41467-022-33106-1 (PMC9470582; doi:10.1038/s41467-022-33106-1)
Supplement: Supplementary file 1 — Supplementary Information [file 41467_2022_33106_MOESM1_ESM.pdf]

Supplementary Information

Enhanced Arctic sea ice melting controlled by larger heat discharge of mid-Holocene rivers

Jiang Dong[1,11], Xuefa Shi[1,2,11*], Xun Gong[3,4,5,6,11*], Anatolii S Astakhov[7], Limin Hu[2,8], Xiting Liu[2,8], Gang Yang[1], Yixuan Wang[9], Yuri Vasilenko[7], Shuqing Qiao[1,2], Alexander Bosin[7], Gerrit Lohmann[6,10]

[1] Key Laboratory of Marine Geology and Metallogeny, First Institute of Oceanography, Ministry of Natural Resources, Qingdao, China.

[2] Laboratory for Marine Geology, Pilot Qingdao National Laboratory for Marine Science and Technology, Qingdao, China.

[3] Institute for Advanced Marine Research, China University of Geosciences, Guangzhou, China

[4] State Key Laboratory of Biogeology and Environmental Geology, Hubei Key Laboratory of Marine Geological Resources, China University of Geosciences, Wuhan 430074, China

[5] Shandong Provincial Key Laboratory of Computer Networks, Qilu University of Technology (Shandong Academy of Sciences), Jinan 250101, China

[6] Alfred-Wegener-Institut Helmholtz-Zentrum für Polar- und Meeresforschung, Bremerhaven, Germany.

[7] V.I.Il'ichev Pacific Oceanological Institute, Far Eastern Branch of Russian Academy of Sciences, Vladivostok, Russia.

[8] Key Laboratory of Submarine Geoscience and Prospecting Techniques, College of Marine Geosciences, Ocean University of China, Qingdao, China.

[9] Key Laboratory of Comprehensive and Highly Efficient Utilization of Salt Lake Resources, Qinghai Institute of Salt Lakes, Chinese Academy of Sciences, Xining, China.

[10] Department of Environmental Physics, University of Bremen, Bremen, Germany.

[11] These authors contributed equally: Jiang Dong, Xuefa Shi, and Xun Gong.

*e-mail: xfshi@fio.org.cn (X.S.), gongxun@cug.edu.cn (X.G.)

The Supplementary Information includes:

1. Supplementary Note 1. The East Siberian Arctic Shelf is an ideal region to assess the effect of river heat discharge on sea ice changes.
2. Supplementary Note 2. The sand sediment fraction can indicate Arctic sea ice changes in the Holocene.
3. Supplementary Note 3. The sedimentation rate is a potential proxy of the Siberian River heat discharge in the Holocene.
4. Supplementary Figures 1 to 13.
5. Supplementary Table 1 Radiocarbon age data for the core sediment.
6. Supplementary Table 2 Optically stimulated luminescence (OSL) dating data for quartz.
7. Supplementary References

Supplementary Note 1. The East Siberian Arctic Shelf is an ideal region to assess the effect of river heat discharge on sea ice changes

Because of the seasonal influence of solar insolation, both Arctic sea ice cover and river runoff show seasonal changes[1-3] (Supplementary Fig. 1). From November to the following February, the solar insolation at 75° N and the Russian pan-Arctic river runoff are approximately zero[2,4], and the Arctic sea ice extent is maximized to ~1.55 × 10^7 km^2 at the beginning of March (Supplementary Fig. 1). In contrast, both the solar insolation (~490 W/m^2) and Russian pan-Arctic river heat energy flux peak (10^{16}–10^{17} J/day) in June and July[3-5]. The Arctic sea ice extent tends to decrease from March and reaches its annual minimum values in September (~6.27 × 10^6 km^2), which lags the annual maximum summer solar insolation by approximately two months (Supplementary Fig. 1).

The East Siberian Arctic Shelf (ESAS) is mainly located in the eastern Arctic and includes shallow-water (≤100 m) areas in the Chukchi Sea, East Siberian Sea, and Laptev Sea (Fig. 1b). Observations suggest that from October to June, the ESAS is completely covered by sea ice and serves as one of the most important areas for the net export of sea ice into the Arctic Ocean in association with ice-rafted debris (IRD)[6-8]. During the other months, the sea ice extent markedly decreases, and the September ESAS has become nearly entirely ice-free in recent decades[1]. Additionally, from June to August, permafrost thawing and precipitation were reinforced along with increased solar insolation and subsequent air and river freshwater temperatures (Supplementary Fig. 1). As a result, Russian pan-Arctic rivers contribute a large amount of freshwater and sediment to the ESAS[2]. Hence, sediment strata on the ESAS without obvious reworking and recycling provide a sensitive record of the evolution of sea ice and river discharge during the Holocene.

Supplementary Note 2. The sand sediment fraction can indicate Arctic sea ice changes in the Holocene

The ESAS sedimentary processes have been significantly influenced by seasonal sea ice throughout the Holocene[9-11], which can be directly recorded in the deposition of IRD. Previous studies have suggested that the sand sediment fraction, which is widely observed in modern floating ice, is an appropriate proxy for IRD to indicate paleo-sea ice evolution[7,8,12]. However, the sand percentage may also be influenced by other factors, i.e., gravity flows, river sediment supply, and hydrodynamic force of oceanic currents. Thus, the indication of the sand percentage in the ESAS region should be discussed.

In the sediment core (LV77-36-1) on the low-gradient ESAS, the absence of an erosional surface and the smooth changes in grain size show that the sand sediment fraction was not transported by gravity flows in the context of similar sedimentary environments throughout the Holocene (Supplementary Figs. 2–4).

At sediment core station LV77-41-1 offshore of the mouth of the Indigirka River, the sedimentary environment is significantly influenced by the Siberian River discharging warm freshwater in May and September, when the river runoff significantly decreases (Supplementary Figs. 1 and 13). Hence, the grain size distribution can largely represent the internal changes of the rivers. During the last 6.0 ka, the sand percentage in core LV77-36-1 was higher than that in core LV77-41-1. In particular, the sand that likely came from high Siberian River discharge did not remarkably trigger a high sand percentage in core LV77-36-1 during 7.0–6.0 ka, when the sedimentation rate was high. In contrast,

the decrease in sand from river discharge contrasted with the increased sand percentage in core LV77-36-1 during the last 2.0 ka. Hence, the increased sand percentage since ~8.2 ka contrasts with the internal changes in the Siberian rivers (LV77-41-1) in the coarse sediment fraction and the decreased sedimentation rate (Supplementary Fig. 5), suggesting that the intensified river sediment supply did not mainly result in a high coarse percentage in core LV77-36-1.

The hydrodynamic force of oceanic currents might not be responsible for the changes in grain size during the Holocene. Based on modern observations, Atlantic water inflows along the shelf slope at water depths between 200 and 800 m[13]. Thus, it cannot impact the grain size in the ESAS region through a direct hydrodynamic process (Fig. 1a). Similarly, the area of influence by subsurface Pacific water inflow is limited to the east of 160° E in the context of recent warming[14]. On the other hand, our calculated sortable silt mean size increased from the mid-Holocene (MH) to the late Holocene (LH) (Supplementary Fig. 6). However, the grain size sorting has become poor as the sand percentage has increased since the MH (Supplementary Fig. 5), which contrasts with the possibly intensifying hydrodynamic transport by the Siberian Coastal Current. Moreover, the nonsignificant correlation between the sortable silt mean size and the percentage suggests that the sortable silt is not sufficiently well sorted to indicate the flow change in the Siberian Coastal Current during the Holocene[15]. In particular, a previous study suggested that sortable silt can also be transported by sea ice based on the grain-size distribution of actual sea ice sediment[16]. Therefore, we argue that the positive correlation between the sortable silt mean size and the sand percentage in core LV77-36-1 is linked to the impact of sea ice on the IRD record (Supplementary Fig. 6).

A previous study suggested that the Siberia-sourced ice-rafted Fe grains recorded in the Beaufort Sea increased rapidly from the MH to the LH[17]. Although this record shows distinct patterns with our sand percentage data during the increase, which possibly resulted from the influence of rapid changes in the Arctic Oscillation on the drift paths[17], both records tended to have high values from 7.5 ka to the present day. Moreover, geochemical records of ice-rafted Fe grains in the Fram Strait also indicate that the East Siberia-sourced IRD increased more during the LH than during the MH, which is transported by sea ice[18]. The most likely explanation for these phenomena is that the IRD exports from the ESAS have increased since the MH. Thus, the sand percentage can be interpreted as a proxy for IRD to indicate sea ice change because sand can be gathered in the anchor ice through suspension freezing with rising frazil ice[7,8,19].

In addition, abundant IRD can be rapidly released by the massive melting of sea ice. During the MH, the low IP_{25} records reveal intensified Arctic sea ice melting in spring and summer[9,10]. By comparison, our IRD value was relatively less absent during the MH than during the LH (Fig. 2b–d). Therefore, the percentage of the sand sediment fraction was dominated by sea ice changes rather than melting and can be traced to the Holocene variations in the seasonal ESAS sea ice.

Supplementary Note 3. The sedimentation rate is a potential proxy of Siberian River heat discharge in the Holocene

The Russian pan-Arctic river heat discharge is jointly determined by the amount of river discharge and river freshwater temperatures[3,20]. In the modern climate, seasonally high solar insolation increases river freshwater temperatures while also increasing the volume of river runoff by

intensifying the early summer thawing of Siberian land snow/ice and permafrost, as well as by river basin precipitation[21-23]. The sedimentation rate in the ESAS region can be directly linked with the river freshwater temperature and river runoff. Solar energy input-driven warm freshwater from rivers and associated permafrost thawing intensify fluvial thermal erosion and the subsequent production of river sediment[2,24-29]. Meanwhile, the increased river runoff in the context of recent global warming favors river sediment transport[2,27-29]. Thus, the coherently increased river runoff and freshwater temperature (Fig. 3) jointly result in a relatively high regional sedimentation rate.

Other factors can influence the regional sedimentation rate but can be excluded. The interior ocean hydrodynamic force can redistribute the input sediment. The low-gradient ESAS is completely covered by sea ice for 9 months (from October to the following June)[1]. Hence, winter ocean dynamics are nearly nonexistent. In the other few months, river runoff and the Siberian Coastal Current dominate the water mass in the ESAS region[2,30,31] (Fig. 1b–c). Although the Siberian Coastal Current can redistribute the input sediment and lead to regional differences in sedimentation rates, sedimentary records and seismic profiles indicate that the high sedimentation rates in the MH and the low sedimentation rates in the LH are not individual station records but regionally recorded in the stratified sediments in the ESAS region, which are located in the area of the significant influence of summer Russian pan-Arctic river-discharged freshwater (Supplementary Figs. 2 and 8). In contrast, throughout the Holocene on the ESAS, the temporal distribution patterns of rare earth elements were similar to those of sediment discharged by the Indigirka River (Supplementary Fig. 7), suggesting that the major sediment source did not significantly change and was mainly from the local Siberian River rather than the Kara Sea, which was transported over a long distance by the Siberian Coastal

Current. Therefore, the sedimentation rate is closely linked to the discharge water input of the Russian pan-Arctic rivers into the ESAS region in the MH (e.g., ref. [32]).

In our study, regional sedimentation rate records reveal a decrease in river discharge from the MH to the LH (Supplementary Fig. 8). This result contrasts with that of the coupled atmosphere-ocean circulation model ECHO-G, in which a more rapid decrease in local net evaporation compared to a decline in moisture due to a relative cooling trend resulted in a slight increase in the Eurasian River discharges from the MH to the LH, followed by a pronounced intensification during the preindustrial era[33]. Wagner et al. (2011) suggested that permafrost is another key factor in changing the terrestrial hydrological cycle, while a permafrost module is absent in their modeling simulations, so more discussion about the impact of permafrost on the pan-Arctic rivers is needed. Our results, which are based on multiple paleoclimate records, indicate that the reinforced permafrost thawing due to the insolation-driven higher summer surface air temperature could generate higher river water discharge during the MH than during the present day (Fig. 2). Therefore, the permafrost thawing process plays an important role in enhancing the river discharge of the Russian pan-Arctic in the MH and overcoming the evaporation change by air temperatures. This result is consistent with our reconstruction of enhanced Russian pan-Arctic river discharge based on the sedimentation rate during the warmer summer conditions during the MH compared to present day conditions.

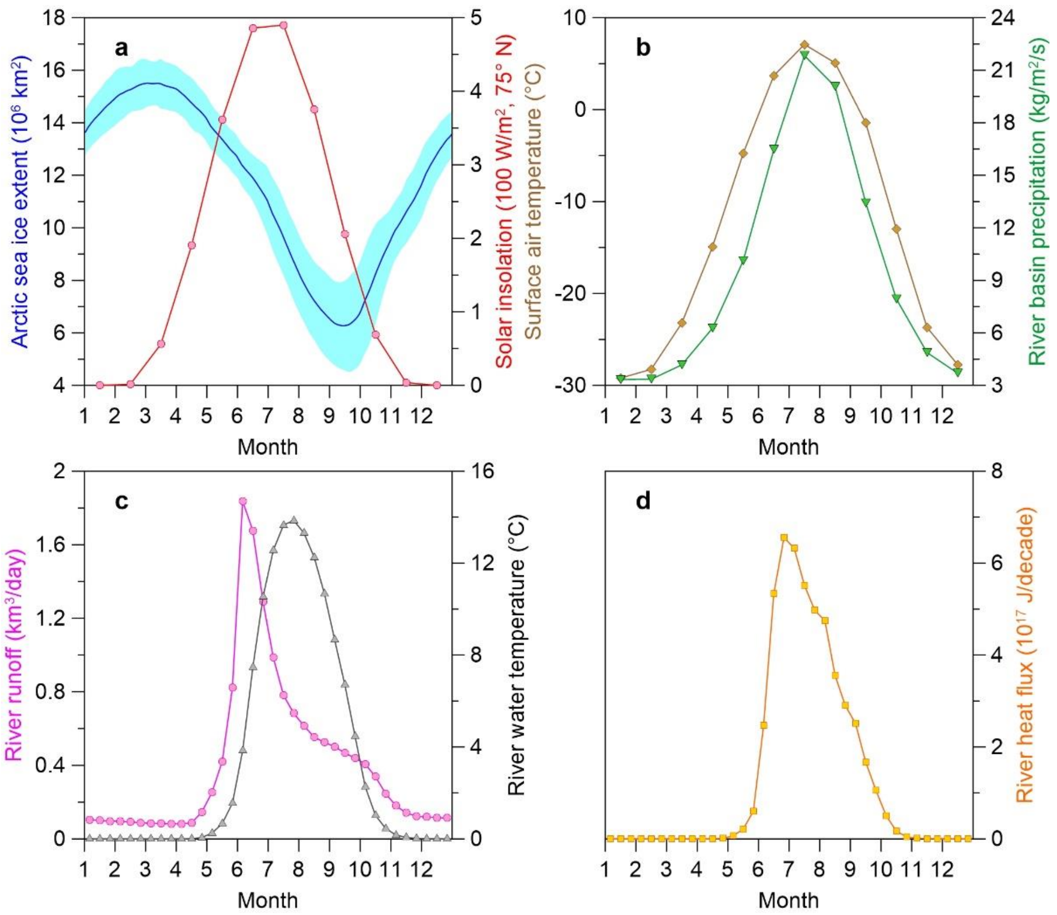

Supplementary Fig. 1 Monthly observations of solar insolation, sea ice extent, river basin precipitation, river discharge, and river freshwater temperature. a Monthly observations of the Arctic sea ice extent with 2-sigma uncertainty (blue area) during 1981–2010 (National Snow and Ice Data Center, https://nsidc.org/arcticseaicenews/) and solar insolation at 75° N[4]. **b** Monthly observations of surface air temperature (85–180° E, 65–80° N) and river basin precipitation (85–180° E, 45–75° N) during 1948–2019 from NCEP/NCAR reanalysis data[34] provided by the NOAA/OAR/ESRL PSL, Boulder, Colorado, USA. **c–d** Monthly records of the Russian pan-Arctic river water discharge, river freshwater temperature, and thermal flux from 1935 to 2001[3]. When the maximum solar insolation occurs in June and July, the Arctic Ocean is largely covered by sea ice, while the Russian pan-Arctic river basin precipitation, river runoff, river freshwater temperature, and heat discharge synchronously peak in early summer (June and July).

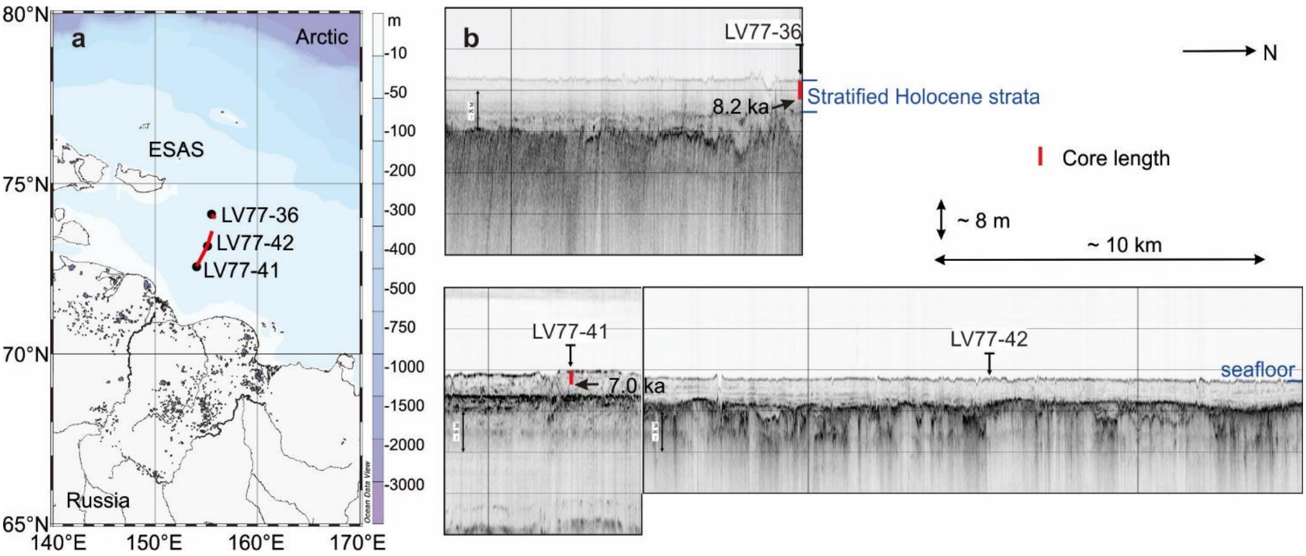

Supplementary Fig. 2 Seismic profiles located on the East Siberian Arctic Shelf (ESAS). a Locations of sediment cores (blue dots) and seismic profiles (red lines). **b** Seismic profiles obtained by the GeoPulse subbottom profiler (GeoAcoustics, Limited, Britain). The surveyed high-resolution seismic profiles were collected by a GeoPulse subbottom profiler (GeoAcoustics, Limited, Britain) to obtain information on the thickness and structure of the upper Quaternary sediment in the ESAS region. The frequency range was from 2 to 12 kHz, which was operated at 3.5 kHz. In the seismic profiles, the widely observed and stratified Holocene strata were well obtained by sediment cores LV77-36-1 and LV77-41-1. Thus, the sedimentary records in this study are representative.

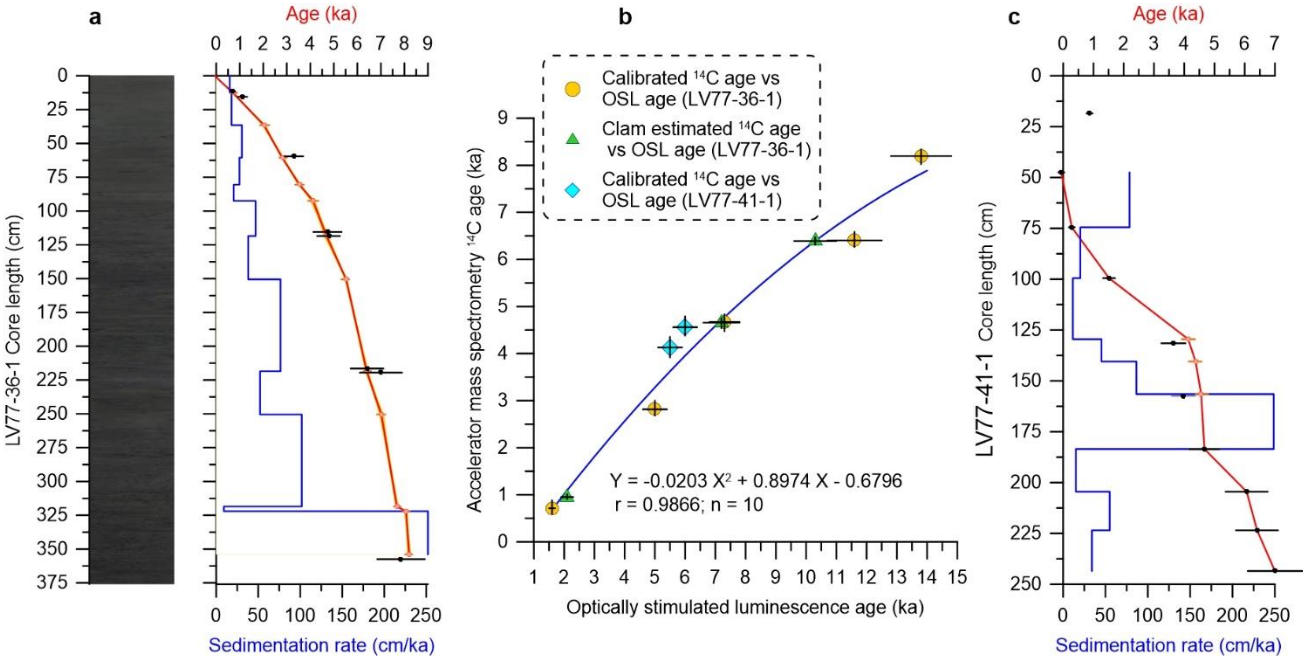

Supplementary Fig. 3 A sediment core photograph and age-depth models. a LV77-36-1 age-depth model. The 2-sigma uncertainty of each age point (short lines) and the age estimations of the best ages (red line) and the related 2-sigma uncertainty (yellow area) are also shown. The age model was reconstructed based on calibrated radiocarbon ages (orange dots and lines). The optically stimulated luminescence (OSL) age points are shown as black dots and lines. **b** Binomial analysis of the data between the radiocarbon ages and the OSL dating of quartz. **c** LV77-41-1 age-depth model based on three calibrated radiocarbon ages (orange dots and lines) and ten recalibrated quartz OSL data (black dots and lines) by the (**b**) linear equation. The related data can be found in Supplementary Tables 1 and 2.

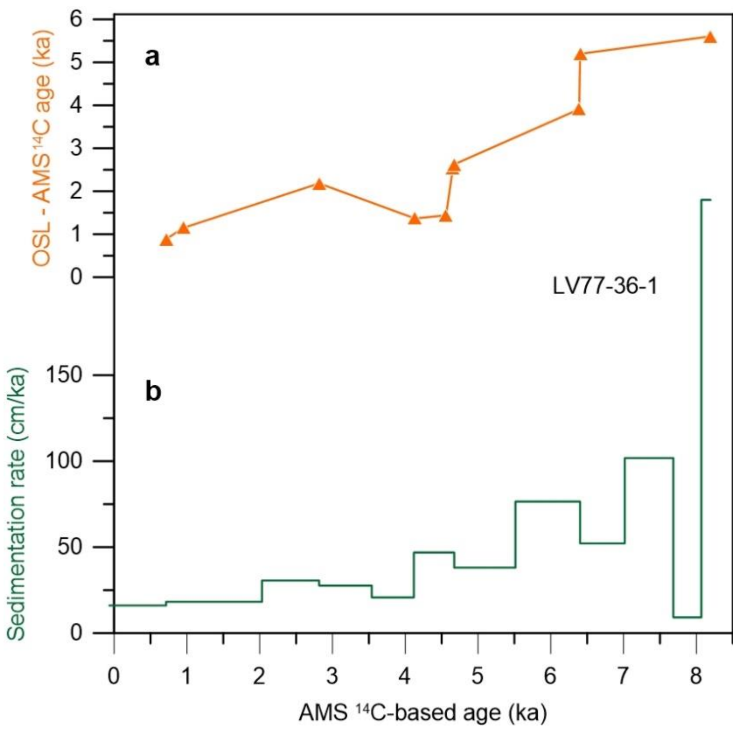

Supplementary Fig. 4 The influence of sedimentation on the optically stimulated luminescence (OSL) age. a Difference values between the OSL age and the accelerator mass spectrometry (AMS) ^{14}C age in core LV77-36-1. **b** Sedimentation rates in core LV77-36-1. The sediment source area has been covered with permafrost and land snow/ice for the long term[21,23], and a large mass of sediment is rapidly transported into the marginal seas due to river erosion mainly in June and July[28,29,35], resulting in high sedimentation rates on the East Siberian Arctic Shelf. All these sedimentary processes prevent the sediment from being exposed to light, leading to the older age estimations by the OSL dating of quartz in the 4–11 μm fraction occurring along with the higher sedimentation rate.

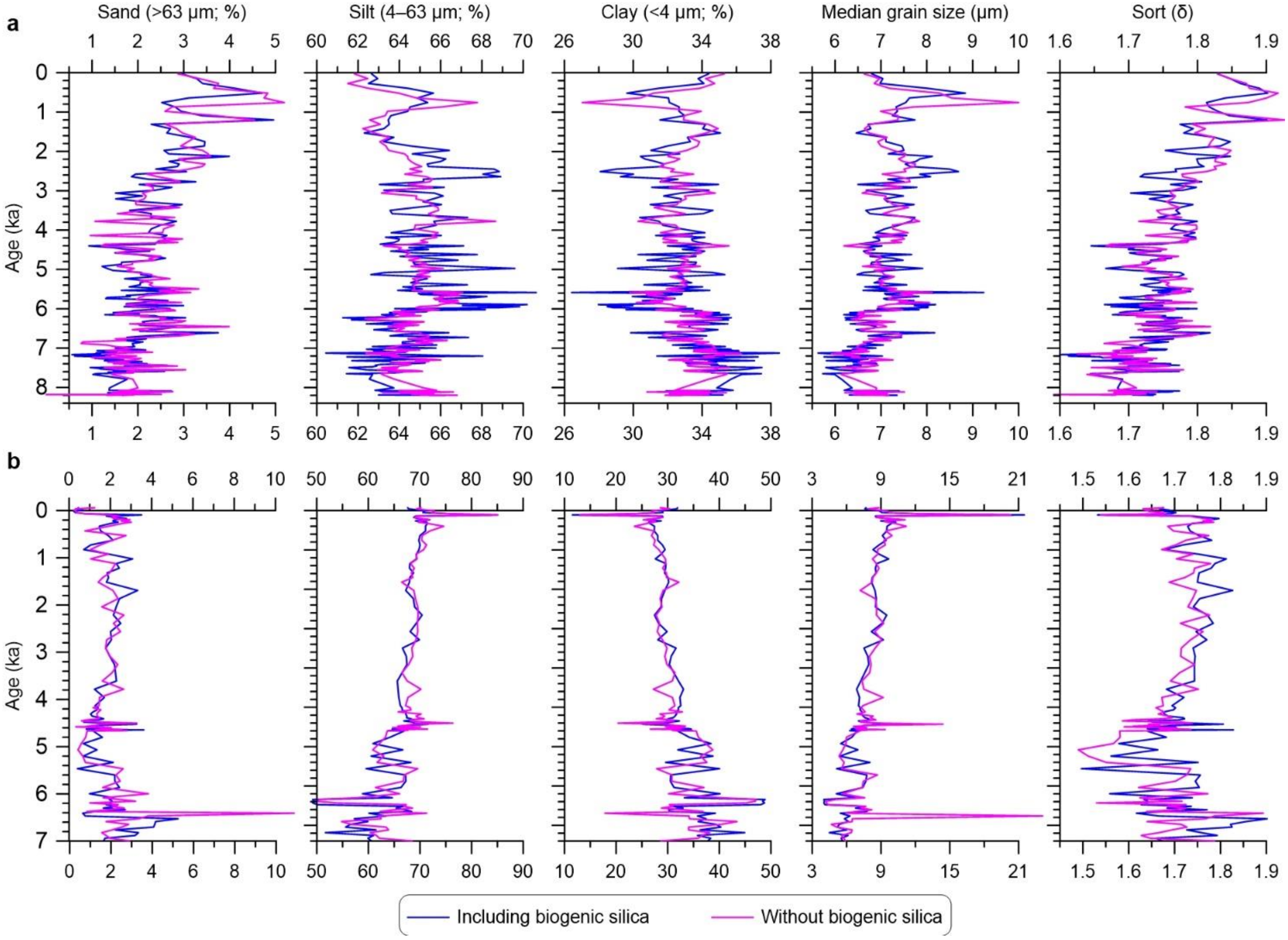

Supplementary Fig. 5 Distributions of the grain-size parameters during the Holocene. a LV77-36-1 located on the East Siberian Arctic Shelf. **b** LV77-41-1 located offshore of the mouth of the Indigirka River. The blue lines are the grain size distributions of sediment, including biogenic silica, while the red lines indicate the grain size distributions of terrigenous material without biogenic silica. The similar trends of parameters between sediments, including and without biogenic silica, suggest that biogenic silica did not significantly change the grain size distribution of terrigenous sediment during the Holocene. Both core sediments mainly consist of silt and subordinate clay, with minor sand throughout the Holocene. The median grain sizes in core LV77-36-1 center on the very fine silt fraction with a range of 5.5–9.0 μm, while the median grain sizes in core LV77-41-1 concentrate in the very fine to fine silt fraction with a range of 3.0–21.0 μm.

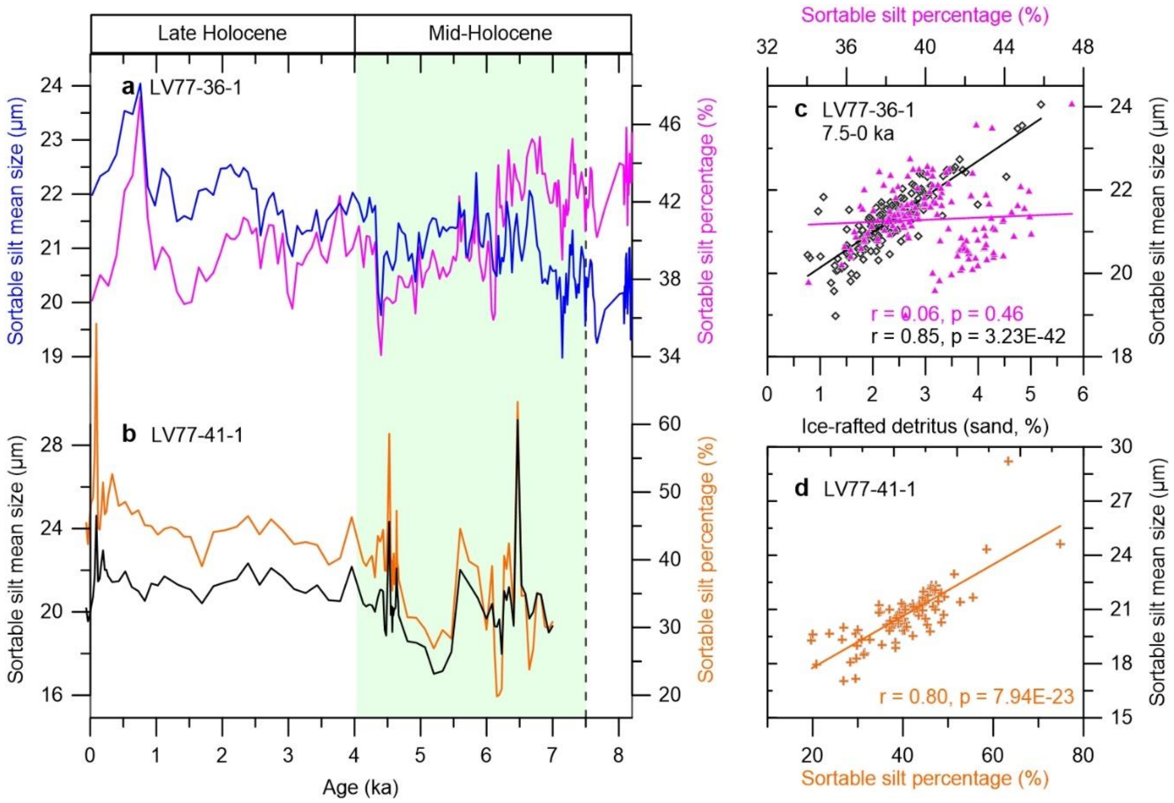

Supplementary Fig. 6 Sortable silt parameters during the Holocene. a–b The sortable silt mean size and percentage in cores LV77-36-1 and LV77-41-1. **c** The sortable silt percentage versus mean size in core LV77-36-1 and the ice-rafted debris (IRD) versus sortable silt mean size. **d** Sortable silt percentage versus mean size in core LV77-41-1. The green band indicates the period from 7.5 to 4.0 ka. The sortable silt mean size in core LV77-36-1 significantly correlates with the IRD rather than the percentage because the developed sea ice can influence the grain size distribution, including the sand, silt, and clay, through rising frazil ice[8,16,19]. Thus, this sortable silt mean size could be influenced by the Siberian Coastal Current, the river water discharge, and sea ice changes. In contrast, the sortable silt mean size in core LV77-41-1 significantly correlates with the percentage, suggesting that the controlling factor of these core sediments is relatively singular, which is probably the river material supply based on modern observations (Figs. 1b–c, Supplementary 13).

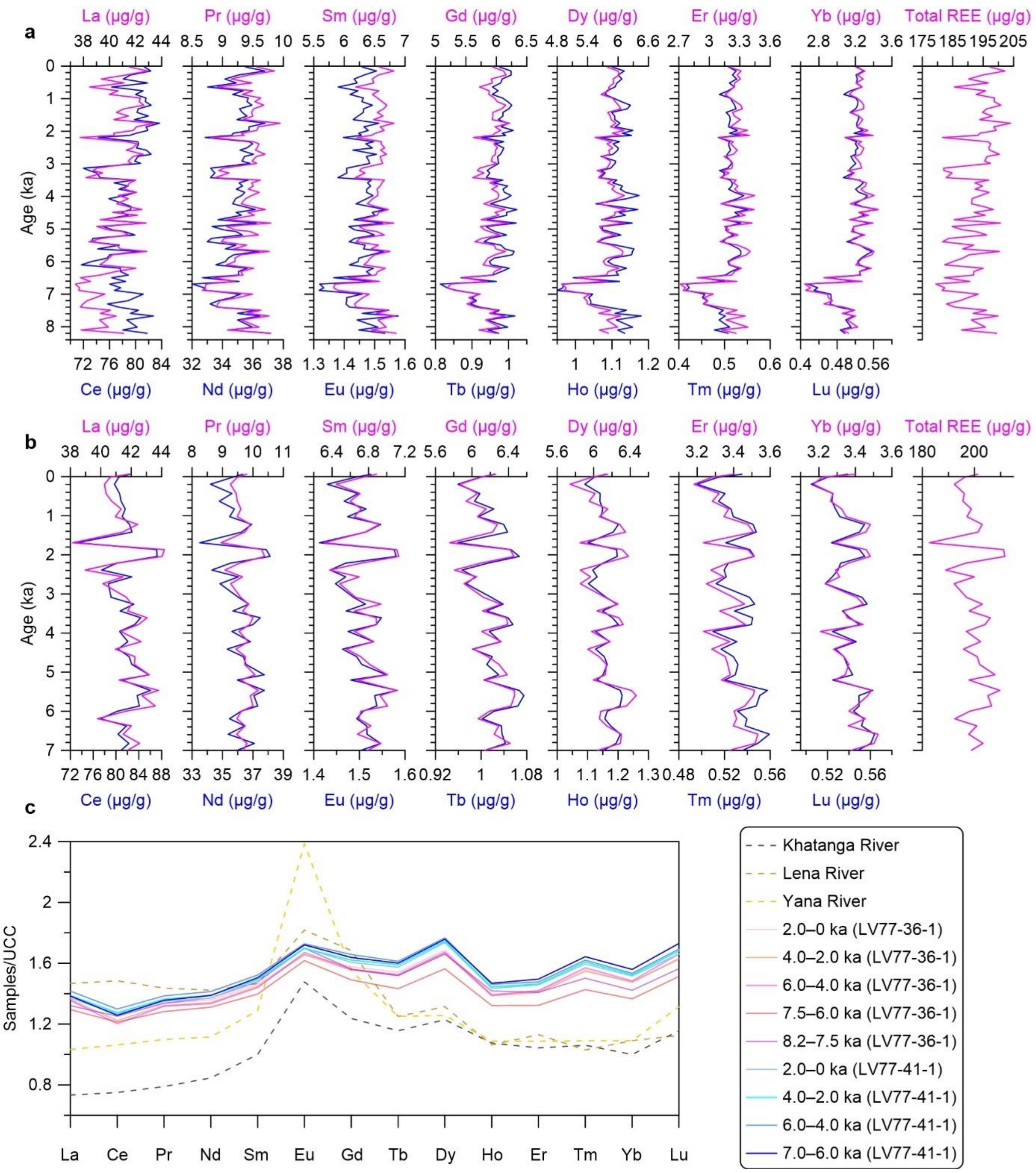

Supplementary Fig. 7 Abundance of rare earth elements in core sediments during the Holocene. a LV77-36-1. **b** LV77-41-1. **c** The upper continental crust (UCC) composite-normalized rare earth elements of the river suspension[36] and in the core sediment. The normalized values are the average of each rare earth element during the given time phase (e.g., 2.0–0 ka) divided by the related value in the UCC[37]. The rare earth element records remain stable, with oscillations throughout the Holocene, suggesting a consistent sediment source in the study area.

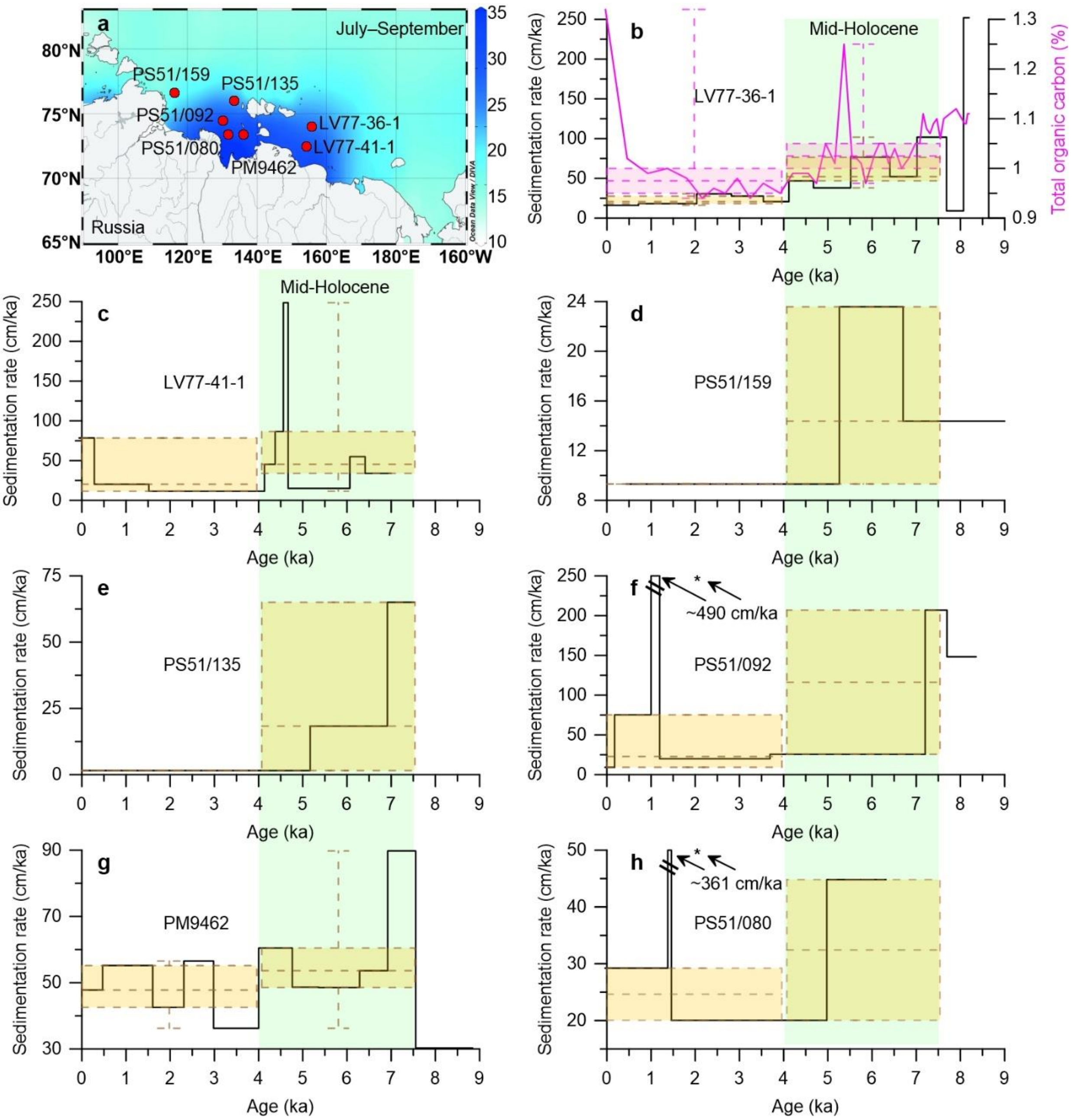

Supplementary Fig. 8 Data compilation of the sedimentation rates in the East Siberian Arctic Shelf region. a Sea surface salinity (PSU) and core locations. **b–h** The sedimentation rate records from the cores of (**b**) LV77-36-1, (**c**) LV77-41-1, (**d**) PS51/159, (**e**) PS51/135, (**f**) PS51/092, (**g**) PM9462, and (**h**) PS51/080[38-40]. In (**b**), the purple curve exhibits the total organic carbon[41]. In addition, the box-whisker plots show the median (middle dotted line), 25th and 75th percentiles (box), the 5th and 95th percentiles (whiskers) and outliers (single points).

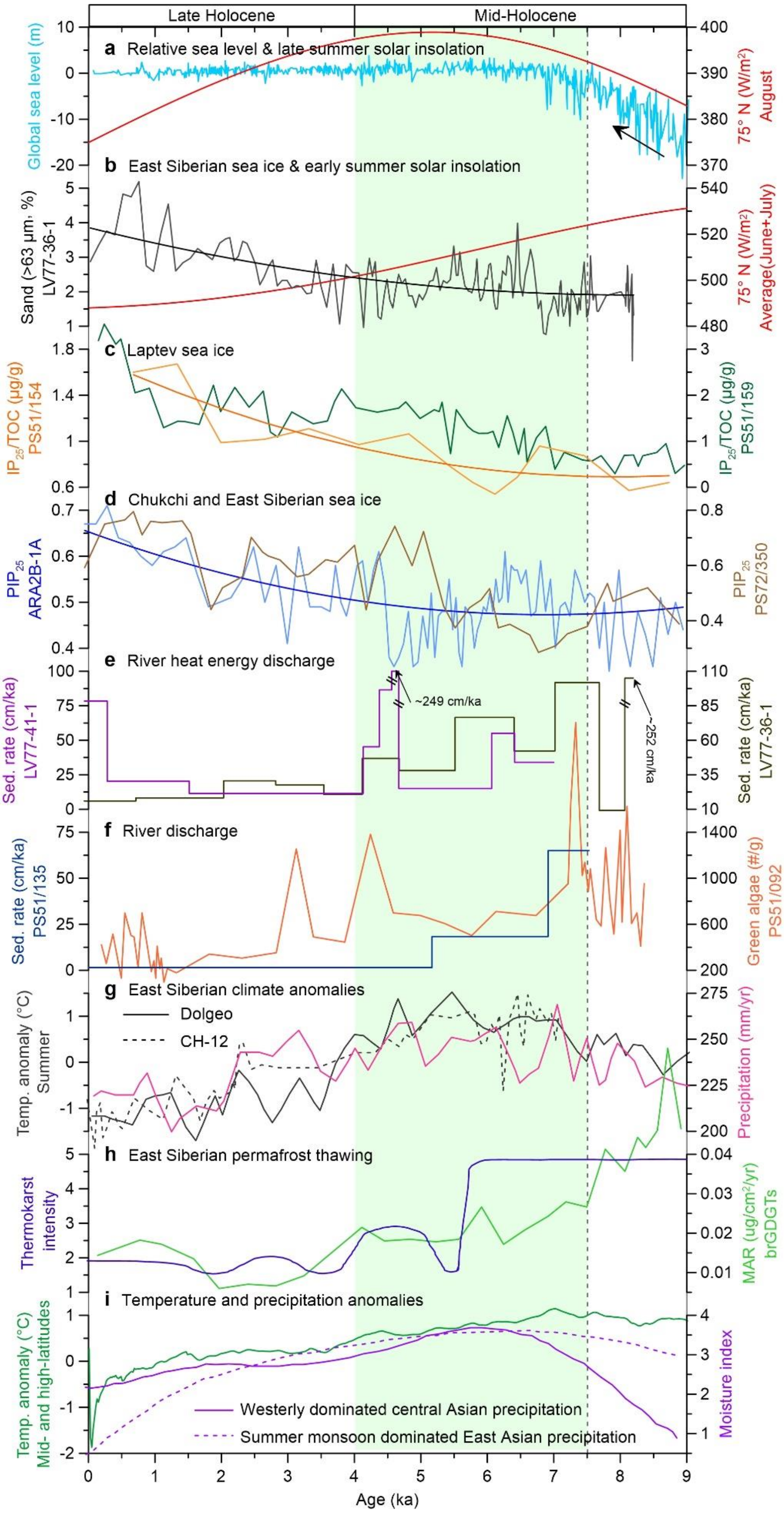

Supplementary Fig. 9 Proxy records for Arctic sea ice and the pan-Arctic river environment during the past 9,000 years. **a** Global sea level[42] and August solar insolation at 75° N[4]. **b** Solar

insolation at 75° N in early summer[4] and reconstructions of the loss of sea ice inferred by low ice-rafted debris in the East Siberian Sea. **c–d** Reconstructions of Arctic sea ice by organic geochemistry from (**c**) the Laptev Sea[10] and (**d**) the Chukchi Sea and East Siberian Sea[9]. The smooth lines represent the binomial expression of the related data to obtain the Holocene orbital tendency. **e** Reconstruction of the Russian pan-Arctic river heat discharge by sedimentation (Sed.) rates. **f** Reconstructions of river discharge by the sedimentation rate in the Laptev Sea[40] and the abundance of freshwater green algae close to the mouth of the Lena River[43]. **g** Pollen-based Russian Arctic anomalies in summer temperature and precipitation[44,45]. **h** Reconstructions of Siberian permafrost thawing by thermokarst lake development[46] and the mass accumulation rate (MAR) of branched glycerol dialkyl glycerol tetraethers (brGDGTs)[21]. **i** Summaries of air temperature in mid- and high-latitude regions[47] and the central and eastern Asian moisture[48] based on hundreds of sedimentary records. During the mid-Holocene (MH), strong summer solar insolation resulted in intensified temperatures in middle and high latitudes[47], and the increased central and eastern Asian moisture was related to enhanced westerlies and the East Asian summer monsoon, respectively[48]. This study focused on the influence of pan-Arctic river heat discharge on Arctic sea ice during the MH from 7.5 to 4.0 ka (green band) because the significant sea-level rise during 9.0–7.5 ka could have reduced the distance between the mouth of the paleo-river and the core locations and thus affected the sedimentation rates.

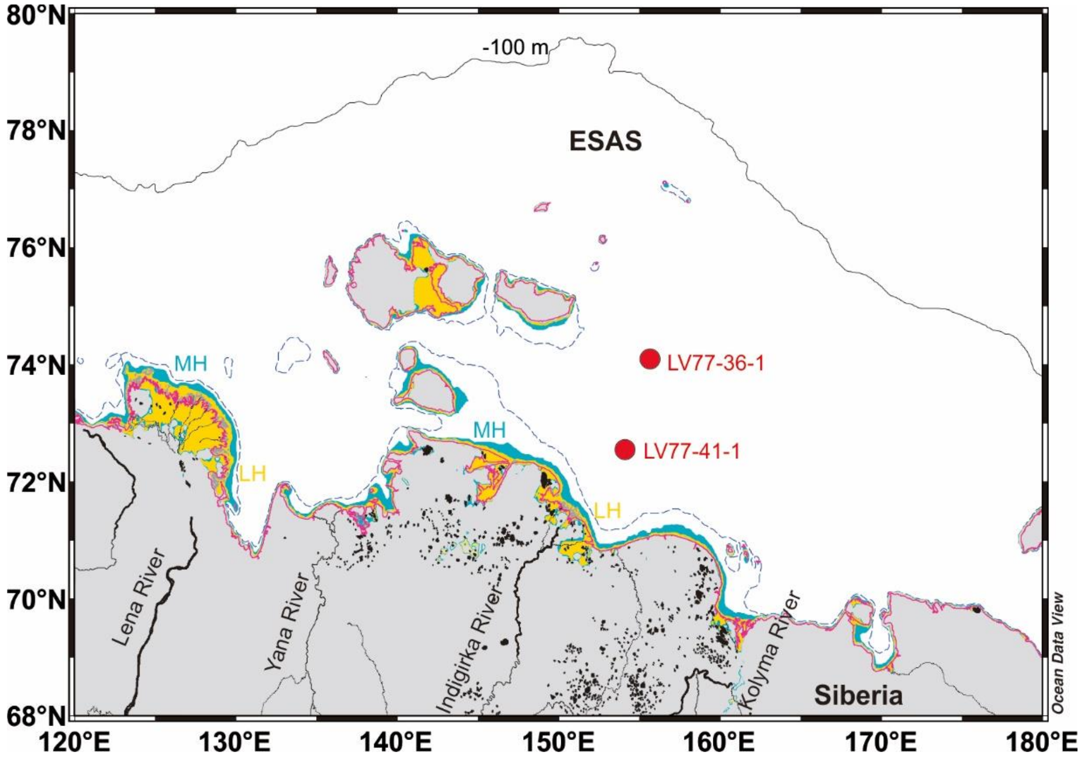

Supplementary Fig. 10 Paleo-sea levels in the East Siberian Arctic Shelf (ESAS) region, according to Lambeck et al. (2014)[42]. The blue and yellow areas show the potential positions of paleo-coastlines during the mid-Holocene (MH) and late Holocene (LH), respectively. The red line is the location of the present-day coastline. The dashed blue line points to the outlier of the reconstructed sea level during the MH.

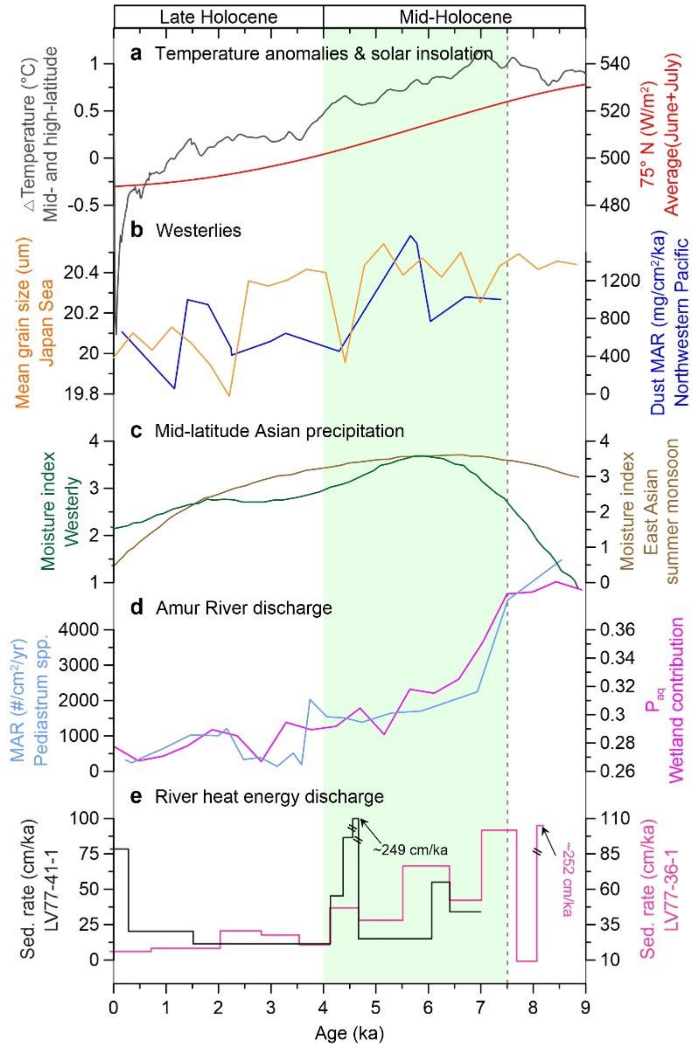

Supplementary Fig. 11 Proxy records for midlatitude climate changes and Russian pan-Arctic river heat discharge. a Solar insolation at 75° N in the early summer[4] and reconstruction of the temperature in the middle and high latitudes[47]. **b** Reconstructions of westerlies by the main grain size of the sensitive silt component[49] and the dust mass accumulation rate (MAR)[50]. **c** Reconstructions of precipitation in midlatitude central and eastern Asia[48]. **d** Reconstructions of the discharge of the Amur River by MAR of Chlorophyceae freshwater algae (*Pediastrum* spp.)[21] and Paq ratio[51]. The Amur River is the northernmost river in the Asian summer monsoon area (Fig. 1a), and the change in the river discharge during the Holocene is consistent with that of the Russian pan-Arctic rivers. **e** Reconstructions of Russian pan-Arctic river heat discharge by sedimentation (Sed.) rates. The green band indicates the period of the mid-Holocene from 7.5 to 4.0 ka.

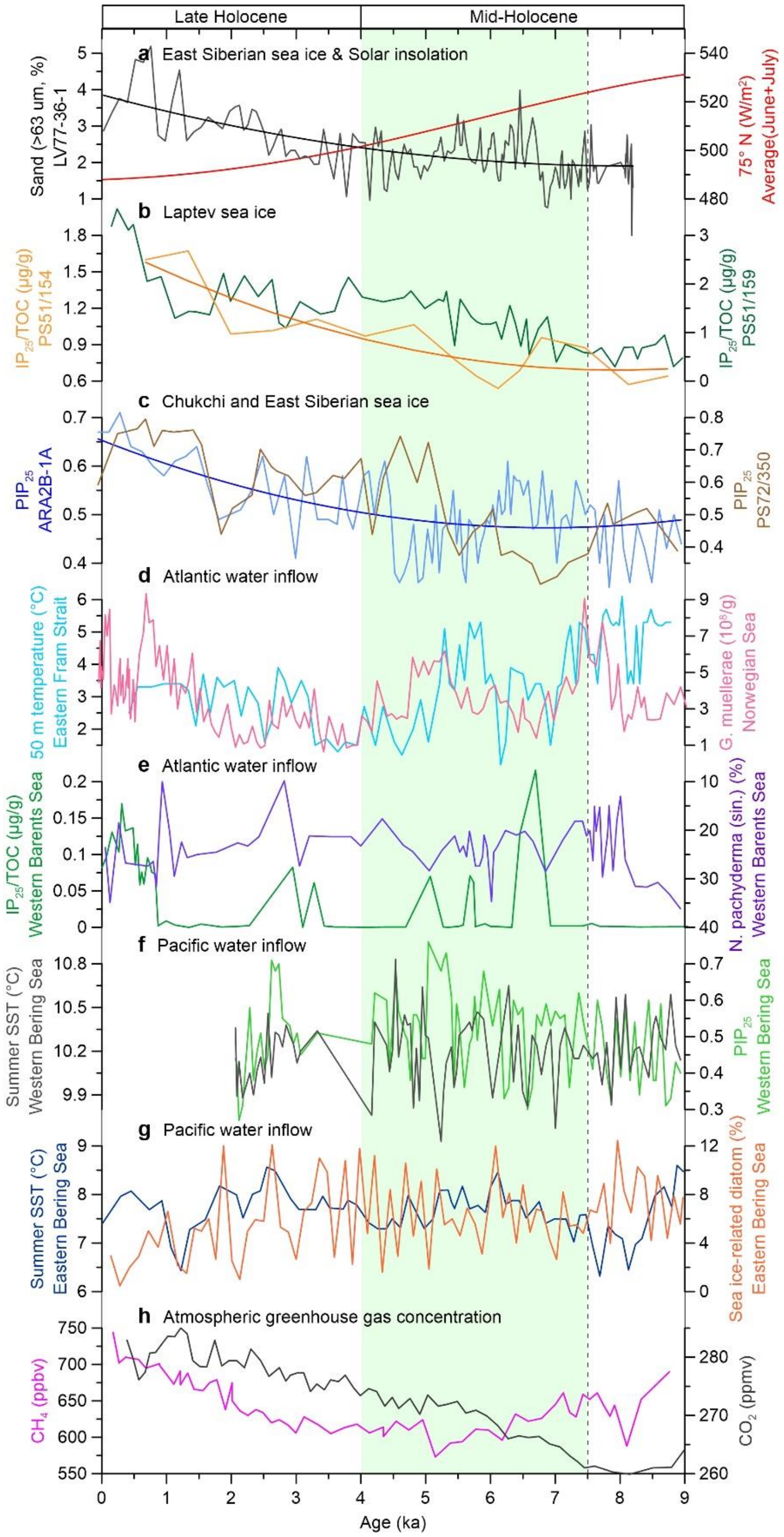

Supplementary Fig. 12 Proxy records for Arctic sea ice, inflows of Atlantic and Pacific warm water, and concentrations of greenhouse gases during the Holocene. a–c Summer insolation at 75° N[4] and reconstructions of Arctic sea ice by (**a**) ice-rafted debris (this study) and organic geochemistry (**b**) IP_{25} (ref. [10]) and (**c**) PIP_{25} (ref. [9]). The smooth lines represent the binomial

expression of the related sea ice proxies to obtain the Holocene orbital tendency. **d–e** Reconstructions of the Atlantic warm water inflow by multiple proxies collected in or near the major axes of the Atlantic water inflows (Fig. 1), including (**d**) summer subsurface (water depth ~50 m) temperature in the eastern Fram Strait[52], coccolith concentration in the Norwegian Sea[53], and (**e**) sea ice and diagnostic foraminiferal species in the western Barents Sea[54]. **f–g** Reconstructions of the Pacific warm water inflow by the summer sea surface temperature (SST) and seasonal sea ice records in the (**f**) western[55] and (**g**) eastern Bering Sea[56]. Biomarkers in the western and eastern Bering Sea, which is located upstream of the Pacific inflow (Fig. 1), show similar conditions of sea ice change and sea surface temperatures, implying a relatively stable state throughout the Holocene[55,56]. **h** Reconstructions of the concentrations of atmospheric methane[57] and carbon dioxide[58]. The low concentrations of both greenhouse gases were opposite to the loss of Arctic sea ice during the mid-Holocene from 7.5 to 4.0 ka (green band).

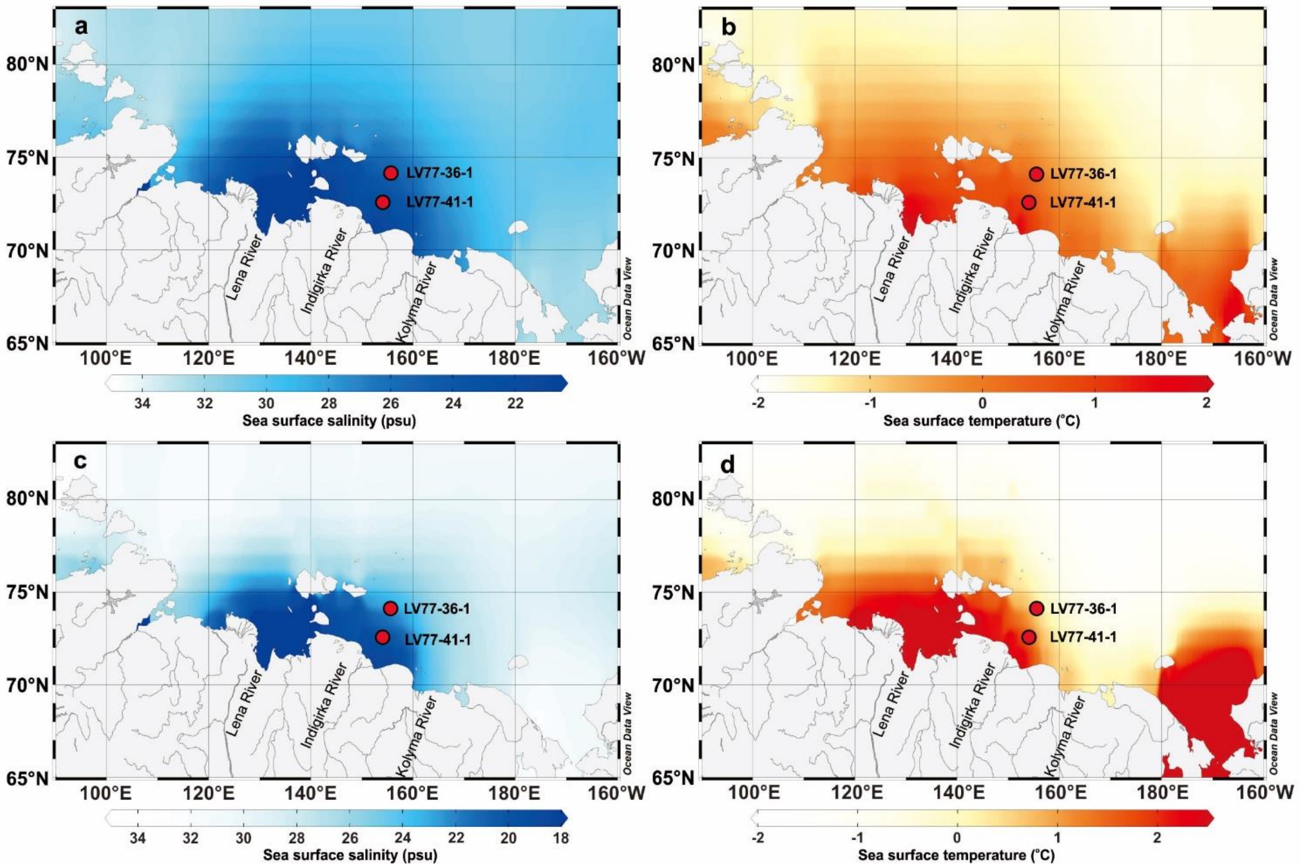

Supplementary Fig. 13 Sea surface salinity and temperature in the East Siberian Arctic Shelf region. a, c The sea surface salinity in the cold (May) and warm (September) months, respectively. **b, d** The sea surface temperature in the cold (May) and warm (September) months, respectively. Datasets were sourced from the World Ocean Atlas 2013 (refs. [59,60]).

Supplementary Table 1 Radiocarbon age data for the core sediment.

Lab. No.	Depth (cm)	Materials	AMS ^{14}C ages (yr BP)	Calibrated ages (cal. yr BP) Before A.D. 1950	2σ (yr BP)	References
**LV77-36-1**						
Beta-478652	12–13	Bivalve shells	1210 ± 30	714	594–884	Ref. [41]*
Beta-478657	36–37	Bivalve shells	2450 ± 30	2032	1858–2255	Ref. [41]*
Beta-478653	60–61	Bivalve shells	3090 ± 30	2817	2682–3001	Ref. [41]*
Beta-478654	80–81	Bivalve shells	3690 ± 30	3540	3357–3727	Ref. [41]*
Beta-478655	92–93	Bivalve shells	4130 ± 30	4118	3903–4345	Ref. [41]*
Beta-520190	118–119	Bivalve shells	4540 ± 30	4673	4473–4839	This study
Beta-520191	150–151	Bivalve shells	5210 ± 30	5514	5318–5650	This study
Beta-520192	218–219	Bivalve shells	6040 ± 30	6403	6263–6586	This study
Beta-520193	250–251	Bivalve shells	6560 ± 30	7016	6824–7199	This study
Beta-520194	318–319	Bivalve shells	7270 ± 30	7684	7551–7841	This study
Beta-466792	322	Bivalve shells	7660 ± 30	8068	7920–8236	Ref. [41]*
Beta-466793	354	Bivalve shells	7780 ± 30	8195	8019–8342	Ref. [41]*
**LV77-41-1**						
Beta-530770	129–130	Bivalve shells	4140 ± 30	4132	3918–4358	This study
Beta-530771	140–141	Bivalve shells	4320 ± 30	4375	4147–4574	This study
Beta-530772	156–157	Bivalve shells	4450 ± 30	4560	4382–4791	This study

The marker (*) represents published raw age data that are recalibrated to calendar years with the new datasets in this study.

Supplementary Table 2 Optically stimulated luminescence (OSL) dating data for quartz.

Lab. No.	Depth (cm)	K (%)	Th (μg/g)	U (μg/g)	Water content (%)	Dose rate Gy/ka)	De (Gy)	OSL age (ka)	Calibrated ages (cal. ka BP) Before A.D. 1950
**LV77-36-1**									
OSL-1A	10–13	2.60 ± 0.04	11.64 ± 0.70	2.33 ± 0.30	44.2 ± 5	2.35 ± 0.16	3.8 ± 0.2	1.6 ± 0.1	0.704 ± 0.1
OSL-1B	14–17	2.61 ± 0.04	11.21 ± 0.70	2.37 ± 0.30	47.5 ± 5	2.19 ± 0.15	4.5 ± 0.2	2.1 ± 0.2	1.115 ± 0.2
OSL-2A	58–61	2.64 ± 0.04	11.73 ± 0.70	2.41 ± 0.30	46.7 ± 5	2.27 ± 0.16	11.5 ± 0.2	5.0 ± 0.4	3.300 ± 0.4
OSL-3A	114–117	2.64 ± 0.04	11.69 ± 0.70	2.46 ± 0.30	44.7 ± 5	2.37 ± 0.16	17.1 ± 0.6	7.2 ± 0.6	4.729 ± 0.6
OSL-3B	117–120	2.65 ± 0.04	11.90 ± 0.70	2.51 ± 0.40	45.9 ± 5	2.33 ± 0.17	17.0 ± 0.3	7.3 ± 0.5	4.789 ± 0.5
OSL-4A	215–218	2.69 ± 0.04	11.72 ± 0.70	2.48 ± 0.30	45.7 ± 5	2.34 ± 0.16	24.0 ± 0.5	10.3 ± 0.7	6.410 ± 0.7
OSL-4B	218–221	2.62 ± 0.04	12.03 ± 0.70	2.57 ± 0.40	51.1 ± 5	2.09 ± 0.15	24.2 ± 0.4	11.6 ± 0.9	6.999 ± 0.9
OSL-5A	356–359	2.54 ± 0.04	11.42 ± 0.70	2.21 ± 0.30	40.4 ± 5	2.45 ± 0.17	33.9 ± 0.9	13.8 ± 1.0	7.839 ± 1.0
**LV77-41-1**									
OSL-1A.1B	17–20	2.49 ± 0.04	12.09 ± 0.70	2.94 ± 0.40	44.0 ± 5	2.45 ± 0.18	4.4 ± 0.2	1.8 ± 0.1	0.870 ± 0.1*
OSL-2A	46–49	2.47 ± 0.04	11.72 ± 0.70	2.76 ± 0.40	54.0 ± 5	1.94 ± 0.14	1.4 ± 0.1	0.7 ± 0.1	-0.061 ± 0.1
OSL-3A.3B	73–76	2.45 ± 0.04	11.64 ± 0.70	2.75 ± 0.40	48.0 ± 5	2.20 ± 0.16	2.3 ± 0.2	1.1 ± 0.1	0.283 ± 0.1
OSL-4A.4B	98–101	2.47 ± 0.04	12.09 ± 0.70	2.89 ± 0.40	49.0 ± 5	2.18 ± 0.16	5.7 ± 0.1	2.6 ± 0.2	1.516 ± 0.2
OSL-5A.5B	130–133	2.59 ± 0.04	12.30 ± 0.70	3.20 ± 0.40	42.0 ± 5	2.62 ± 0.19	14.4 ± 0.1	5.5 ± 0.4	3.642 ± 0.4
OSL-6C	156–159	2.52 ± 0.04	12.24 ± 0.70	2.95 ± 0.40	39.0 ± 5	2.70 ± 0.20	16.1 ± 0.2	6.0 ± 0.4	3.974 ± 0.4
OSL-7C.7D	182–185	2.64 ± 0.04	12.00 ± 0.70	3.01 ± 0.40	46.0 ± 5	2.41 ± 0.17	17.1 ± 0.3	7.1 ± 0.5	4.669 ± 0.5
OSL-8C.8D	203–206	2.52 ± 0.04	11.80 ± 0.70	3.06 ± 0.40	42.0 ± 5	2.57 ± 0.18	24.5 ± 0.6	9.6 ± 0.7	6.065 ± 0.7
OSL-9C.9D	222–225	2.49 ± 0.04	11.82 ± 0.70	2.89 ± 0.40	51.0 ± 5	2.08 ± 0.15	21.3 ± 0.3	10.3 ± 0.7	6.410 ± 0.7
OSL-10C	242–245	2.55 ± 0.04	10.72 ± 0.70	3.05 ± 0.40	48.0 ± 5	2.24 ± 0.16	26.0 ± 0.4	11.6 ± 0.9	6.999 ± 0.9

The reversed age (marker *) indicates that the sediment may be reworked and recycled, but this phenomenon cannot be observed in the sedimentary facies. Thus, this study focused on the sedimentary records between 245 and 46 cm in core LV77-41-1.

References

1 Comiso, J. C., Parkinson, C. L., Gersten, R. & Stock, L. Accelerated decline in the Arctic sea ice cover. *Geophys. Res. Lett.* **35,** L01703 (2008).

2 Milliman, J. D. & Farnsworth, K. L. *River Discharge to the Coastal Ocean: A Global Synthesis* (Cambridge Univ. Press, New York, 2013).

3 Lammers, R. B., Pundsack, J. W. & Shiklomanov, A. I. Variability in river temperature, discharge, and energy flux from the Russian pan-Arctic landmass. *J. Geophys. Res.: Biogeosci.* **112,** G04S59 (2007).

4 Laskar, J. et al. A long-term numerical solution for the insolation quantities of the Earth. *Astron. Astrophys.* **428,** 261-285 (2004).

5 Perovich, D. K., Nghiem, S. V., Markus, T. & Schweiger, A. Seasonal evolution and interannual variability of the local solar energy absorbed by the Arctic sea ice–ocean system. *J. Geophys. Res.* **112,** C03005 (2007).

6 Krumpen, T. et al. Variability and trends in Laptev Sea ice outflow between 1992–2011. *Cryosphere* **7,** 349-363 (2013).

7 Darby, D. A., Myers, W. B., Jakobsson, M. & Rigor, I. Modern dirty sea ice characteristics and sources: The role of anchor ice. *J. Geophys. Res.* **116,** C09008 (2011).

8 Nürnberg, D. et al. Sediments in Arctic sea ice: Implications for entrainment, transport and release. *Mar. Geol.* **119,** 185-214 (1994).

9 Stein, R. et al. Holocene variability in sea ice cover, primary production, and Pacific-Water inflow and climate change in the Chukchi and East Siberian Seas (Arctic Ocean). *J. Quaternary Sci.* **32,** 362-379 (2017).

10 Hörner, T., Stein, R., Fahl, K. & Birgel, D. Post-glacial variability of sea ice cover, river run-off and biological production in the western Laptev Sea (Arctic Ocean) – A high-resolution biomarker study. *Quaternary Sci. Rev.* **143,** 133-149 (2016).

11 Farmer, J. R. et al. Western Arctic Ocean temperature variability during the last 8000 years. *Geophys. Res. Lett.* **38,** L24602 (2011).

12 Polyak, L. et al. History of sea ice in the Arctic. *Quaternary Sci. Rev.* **29,** 1757-1778 (2010).

13 Carmack, E. et al. Toward Quantifying the Increasing Role of Oceanic Heat in Sea Ice Loss in the New Arctic. *B. Am. Meteorol. Soc.* **96,** 2079-2105 (2015).

14 Semiletov, I. The East Siberian Sea as a transition zone between Pacific-derived waters and Arctic shelf waters. *Geophys. Res. Lett.* **32,** L10614 (2005).

15 McCave, I. N. & Andrews, J. T. Distinguishing current effects in sediments delivered to the ocean by ice. I. Principles, methods and examples. *Quaternary Sci. Rev.* **212,** 92-107 (2019).

16 Darby, D. A. et al. The role of currents and sea ice in both slowly deposited central Arctic and rapidly deposited Chukchi–Alaskan margin sediments. *Global Planet. Change* **68,** 58-72 (2009).

17 Darby, D. A., Ortiz, J. D., Grosch, C. E. & Lund, S. P. 1,500-year cycle in the Arctic Oscillation identified in Holocene Arctic sea-ice drift. *Nat. Geosci.* **5,** 897-900 (2012).

18 Darby, D. A., Bischof, J. F., Spielhagen, R. F., Marshall, S. A. & Herman, S. W. Arctic ice export events and their potential impact on global climate during the late Pleistocene. *Paleoceanography* **17,** 15-11-15-17 (2002).

19 Reimnitz, E., Clayton, J. R., Kempema, E. W., Payne, J. R. & Weber, W. S. Interaction of rising frazil with suspended panicles: tank experiments with applications to nature. *Cold Reg. Sci.*

Technol. **21,** 117-135 (1993).

20 Yang, D., Shrestha, R. R., Lung, J. L. Y., Tank, S. & Park, H. Heat flux, water temperature and discharge from 15 northern Canadian rivers draining to Arctic Ocean and Hudson Bay. *Global Planet. Change* **204,** 103577 (2021).

21 Winterfeld, M. et al. Deglacial mobilization of pre-aged terrestrial carbon from degrading permafrost. *Nat. Commun.* **9,** 3666 (2018).

22 Zhang, X. et al. Enhanced poleward moisture transport and amplified northern high-latitude wetting trend. *Nat. Clim. Change* **3**, 47-51 (2012).

23 Yang, D. et al. Siberian Lena River hydrologic regime and recent change. *J. Geophys. Res.: Atmos.* **107(D23),** 107, 4694 (2002).

24 Costard, F. et al. Impact of the global warming on the fluvial thermal erosion over the Lena River in Central Siberia. *Geophys. Res. Lett.* **34,** L14501 (2007).

25 Randriamazaoro, R., Dupeyrat, L., Costard, F. & Gailhardis, E. C. Fluvial thermal erosion: heat balance integral method. *Earth Surf. Proc. Land.* **32,** 1828-1840 (2007).

26 Séjourné, A. et al. Evolution of the banks of thermokarst lakes in Central Yakutia (Central Siberia) due to retrogressive thaw slump activity controlled by insolation. *Geomorphology* **241,** 31-40 (2015).

27 Georgiadi, A. G., Kashutina, E. A. & Milyukova, I. P. Long-term Changes of Water Flow, Water Temperature and Heat Flux of the Largest Siberian Rivers. *Polarforschung* **87,** 167-176 (2017).

28 Costard, F., Gautier, E., Fedorov, A., Konstantinov, P. & Dupeyrat, L. An Assessment of the Erosion Potential of the Fluvial Thermal Process during Ice Breakups of the Lena River (Siberia). *Permafrost Periglac. Proc.* **25,** 162-171 (2014).

29 Tananaev, N. I. Hydrological and sedimentary controls over fluvial thermal erosion, the Lena River, central Yakutia. *Geomorphology* **253,** 524-533 (2016).

30 Williams, W. J. & Carmack, E. C. The 'interior' shelves of the Arctic Ocean: Physical oceanographic setting, climatology and effects of sea-ice retreat on cross-shelf exchange. *Prog. Oceanogr.* **139,** 24-41 (2015).

31 Weingartner, T. J., Danielson, S., Sasaki, Y., Pavlov, V. & Kulakov, M. The Siberian Coastal Current: A wind- and buoyancy-forced Arctic coastal current. *J. Geophys. Res.: Oceans* **104,** 29697-29713 (1999).

32 Dong, J. et al. Holocene Climate Modulates Mud Supply, Transport, and Sedimentation on the East China Sea Shelf. *J. Geophys. Res.: Earth Surface* **125,** e2020JF005731 (2020).

33 Wagner, A., Lohmann, G. & Prange, M. Arctic river discharge trends since 7 ka BP. *Global Planet. Change* **79,** 48-60 (2011).

34 Kalnay, E. et al. The NCEP/NCAR 40-year reanalysis project. *B. Am. Meteorol. Soc.* **77,** 437-471 (1996).

35 Magritsky, D., Alexeevsky, N., Aybulatov, D., Fofonova, V. & Gorelkin, A. Features and evaluations of spatial and temporal changes of water runoff, sediment yield and heat flux in the Lena River delta. *Polarforschung* **87,** 89-109 (2017).

36 Rachold, V. Major, trace and rare earth element geochemistry of suspended particulate material of East Siberian rivers draining to the Arctic Ocean. In: Kassens, H., Bauch, H.A., Dmitrenko, I., Eicken, H., Hubberten, H.W., Melles, M., Tiede, J. & Timokhov, L. (Eds.), *Land-Ocean Systems in the Siberian Arctic: Dynamics and History* (Springer-Verlag, Berlin, 1999).

37 Taylor, S. R. & McLennan, S. M. The geochemical evolution of the continental crust. *Rev.*

Geophys. **33,** 241-265 (1995).

38 Wegner, C. et al. Variability in transport of terrigenous material on the shelves and the deep Arctic Ocean during the Holocene. *Polar. Res.* **34,** 24964 (2015).

39 Bauch, H. A. & Polyakova, Y. I. Diatom-inferred salinity records from the Arctic Siberian Margin: Implications for fluvial runoff patterns during the Holocene. *Paleoceanography* **18,** 1027 (2003).

40 Bauch, H. A. et al. Chronology of the Holocene transgression at the North Siberian margin. *Global Planet. Change* **31,** 125-139 (2001).

41 Astakhov, A. S. et al. Distribution and sources of rare earth elements in sediments of the Chukchi and East Siberian Seas. *Polar. Res.* **20,** 148-159 (2019).

42 Lambeck, K., Rouby, H., Purcell, A., Sun, Y. Y. & Sambridge, M. Sea level and global ice volumes from the Last Glacial Maximum to the Holocene. *Proc. Natl. Acad. Sci. U. S. A.* **111,** 15296-15303 (2014).

43 Polyakova, Y. I., Klyuvitkina, T. S., Novichkova, E. A., Bauch, H. A. & Kassens, H. Changes in the Lena River runoff during the Holocene. *Water Resour.* **36,** 273-283 (2009).

44 Klemm, J., Herzschuh, U. & Pestryakova, L. A. Vegetation, climate and lake changes over the last 7000 years at the boreal treeline in north-central Siberia. *Quaternary Sci. Rev.* **147,** 422-434 (2016).

45 Klemm, J. et al. A pollen-climate transfer function from the tundra and taiga vegetation in Arctic Siberia and its applicability to a Holocene record. *Palaeogeogr. Palaeoclimatol. Palaeoecol.* 702-713 (2013).

46 Morgenstern, A. et al. Evolution of thermokarst in East Siberian ice-rich permafrost: A case

study. *Geomorphology* **201,** 363-379 (2013).

47 Marcott, S. A., Shakun, J. D., Clark, P. U. & Mix, A. C. A reconstruction of regional and global temperature for the past 11,300 years. *Science* **339,** 1198-1201 (2013).

48 Chen, F. et al. Westerlies Asia and monsoonal Asia: Spatiotemporal differences in climate change and possible mechanisms on decadal to sub-orbital timescales. *Earth-Sci. Rev.* **192,** 337-354 (2019).

49 Dong, Z. et al. Evolution of westerly jet during the last 60 ka: Evidence from core deposits in the central Japan (East) Sea. *Chinese Science Bulletin* **62,** 1172-1184 (2017).

50 Rea, D. K. & Leinen, M. Asian aridity and the zonal westerlies: Late Pleistocene and Holocene record of eolian deposition in the northwest Pacific Ocean. *Palaeogeogr. Palaeocl.* **66,** 1-8 (1988).

51 Ficken, K. J., Li, B., Swain, D. L. & Eglinton, G. An n-alkane proxy for the sedimentary input of submerged/floating freshwater aquatic macrophytes. *Org. Geochem.* **31,** 745-749 (2000).

52 Werner, K., Spielhagen, R. F., Bauch, D., Hass, H. C. & Kandiano, E. Atlantic Water advection versus sea-ice advances in the eastern Fram Strait during the last 9 ka: Multiproxy evidence for a two-phase Holocene. *Paleoceanography* **28,** 283-295 (2013).

53 Giraudeau, J. et al. Millennial-scale variability in Atlantic water advection to the Nordic Seas derived from Holocene coccolith concentration records. *Quaternary Sci. Rev.* **29,** 1276-1287 (2010).

54 Berben, S. M. P., Husum, K., Cabedo-Sanz, P. & Belt, S. T. Holocene sub-centennial evolution of Atlantic water inflow and sea ice distribution in the western Barents Sea. *Clim. Past.* **10,** 181-198 (2014).

55 Ruan, J. et al. Holocene variability in sea surface temperature and sea ice extent in the northern Bering Sea: A multiple biomarker study. *Org. Geochem.* **113,** 1-9 (2017).

56 Harada, N. et al. Holocene sea surface temperature and sea ice extent in the Okhotsk and Bering Seas. *Prog. Oceanogr.* **126,** 242-253 (2014).

57 Blunier, T., Chappellaz, J. A., Schwander, J., Stauffer, B. & Raynaud, D. Variations in atmospheric methane concentration during the Holocene epoch. *Nature* **374,** 46-49 (1995).

58 Indermühle, A. et al. Holocene carbon-cycle dynamics based on CO_2 trapped in ice at Taylor Dome, Antarctica. *Nature* **398,** 121-126 (1999).

59 Locarnini, R. A. et al. World Ocean Atlas 2013, Volume 1: Temperature. National Oceanographic Data Center, http://www.nodc.noaa.gov/OC5/indprod.html (2013).

60 Zweng, M. M. et al. World Ocean Atlas 2013, Volume 2: Salinity. National Oceanographic Data Center, http://www.nodc.noaa.gov/OC5/indprod.html (2013).
